# Supplementary material for: Fecal Microbiota Transplant in a Pre-Clinical Model of Type 2 Diabetes Mellitus, Obesity and Diabetic Kidney Disease
Source: Int J Mol Sci. 2022 Mar 31;23(7):3842. doi: 10.3390/ijms23073842 (PMC8998923; doi:10.3390/ijms23073842)
Supplement: Supplementary file 1 [file ijms-23-03842-s001.zip › ijms-1580461-supplementary.pdf]

SUPPLEMENTARY MATERIAIS

RESULTS

Analysis of the sequencing of the 16S rRNA

In all groups, most sequences revealed greater proportions of the *Bacteroidetes* phylum: 10-week-old BTBR WT (60.33%), 10-week-old BTBR$^{ob/ob}$ (71.36%), 14-week-old BTBR WT (60.70%), 14-week-old BTBR FMT (-) (74.01%), and 14-week-old BTBR FMT (+) (73.44%) mice when compared to the *Firmicutes* phylum*:* 10-week-old BTBR WT (24.10%), 10-week-old BTBR$^{ob/ob}$ (16.38%), 14-week-old BTBR WT (26.28%), 14-week-old BTBR FMT (-) (14.36%), and 14-week-old BTBR FMT (+) (18.78%) mice.

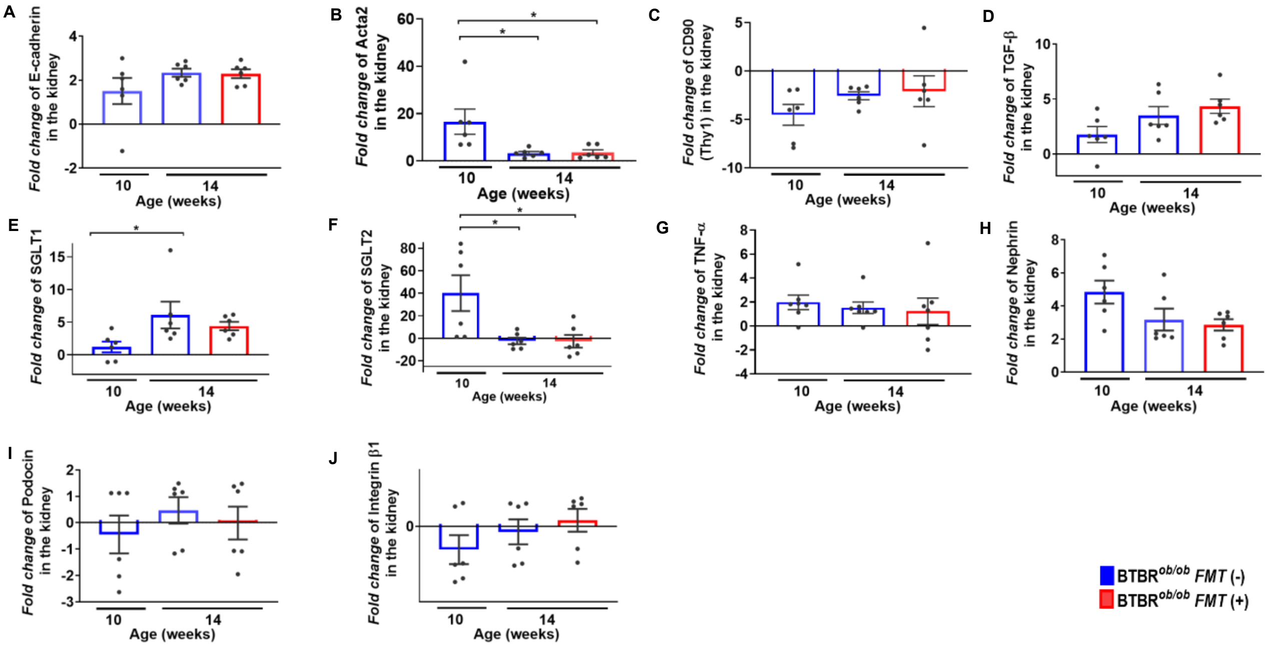

Supplementary Figure S1 . Gene expression of tubulointerstitial, podocyte, mesangial and inflammatory markers by qPCR in the kidney in BTBR$^{ob/ob}$ FMT (+) and BTBR$^{ob/ob}$ FMT (-) mice in relation to BTBR WT mice. (**A**) Gene expression of E-cadherin in the kidney of 10-week-old BTBR$^{ob/ob}$ *versus* 14-week-old BTBR$^{ob/ob}$ FMT (-) mice was not different (*P*=0.29) and *versus* 14-week-old BTBR$^{ob/ob}$ FMT (+) (*P*=0.33) mice, and between 14-week-old BTBR$^{ob/ob}$ FMT (+) and 14-week-old BTBR$^{ob/ob}$ FMT (-) (*P*>0.99) mice. (**B**) Gene expression of alpha actin (Acta2) in the kidney of 10-week-old BTBR$^{ob/ob}$ *versus* 14-week-old BTBR$^{ob/ob}$ FMT (-) mice was significantly different (**P*=0.024) and *versus* 14-week-old BTBR$^{ob/ob}$ FMT (+) (**P*=0.033) mice. No significant difference was found between 14-week-old BTBR$^{ob/ob}$ FMT (-) *versus* 14-week-old BTBR$^{ob/ob}$ FMT (+) (*P*>0.99) mice. (**C**) Gene expression of Thy-1 (CD90) did not change between BTBR$^{ob/ob}$ mice in accordance with time and treatment: 10-week-old BTBR$^{ob/ob}$ *versus* 14-week-old BTBR$^{ob/ob}$ FMT (-) (*P*=0.45) and 14-week-old BTBR$^{ob/ob}$ FMT (+) (*P*=0.31) mice, and 14-week-old BTBR$^{ob/ob}$ FMT (-) *versus* 14-week-old BTBR$^{ob/ob}$ FMT (+) (*P*=0.95) mice. (**D**) Gene expression of TGF-β in the kidney did not significantly change between 10-week-old BTBR$^{ob/ob}$ *versus* 14-week-old BTBR$^{ob/ob}$ FMT (-) (*P*=0.25) and 14-week-old BTBR$^{ob/ob}$ FMT (+) (*P*=0.06) mice, and between 14-week-old BTBR$^{ob/ob}$ FMT (+) *versus* 14-week-old BTBR$^{ob/ob}$ FMT (-) (*P*=0.70) mice. (**E**) Gene expression of SGLT1 in the kidney of 10-week-old BTBR$^{ob/ob}$ *versus* 14-week-old BTBR$^{ob/ob}$ FMT (-) mice was significantly different (**P*=0.033), but not with 14-week-old BTBR$^{ob/ob}$ FMT (+) (*P*=0.06) mice, and between 14-week-old BTBR$^{ob/ob}$ FMT (-) and 14-week-old BTBR$^{ob/ob}$ FMT (+) (*P*>0.99) mice. (**F**) Gene expression of SGLT2 in the kidney was significantly different among 10-week-old BTBR$^{ob/ob}$ *versus* 14-week-old BTBR$^{ob/ob}$ FMT (-) (**P*=0.021) and 14-week-old BTBR$^{ob/ob}$ FMT (+) (**P*=0.020) mice. No significant difference

was found between 14-week-old BTBR$^{ob/ob}$ FMT (-) *versus* 14-week-old BTBR$^{ob/ob}$ FMT (+) (*P*=0.99) mice. (**G**) Gene expression of TNF-α in the kidney did not significantly change among 10-week-old BTBR$^{ob/ob}$ *versus* 14-week-old BTBR$^{ob/ob}$ FMT (-) (*P*=0.91) and 14-week-old BTBR$^{ob/ob}$ FMT (+) (*P*=0.78) mice, and between 14-week-old BTBR$^{ob/ob}$ FMT (-) and 14-week-old BTBR$^{ob/ob}$ FMT (-) (*P*=0.96) mice. (**H**) Gene expression of nephrin in the kidney showed no significant difference among 10-week-old BTBR$^{ob/ob}$ *versus* 14-week-old BTBR$^{ob/ob}$ FMT (-) (*P*=0.19) and 14-week-old BTBR$^{ob/ob}$ FMT (+) (*P*=0.08) mice, and between 14-week-old BTBR$^{ob/ob}$ FMT (-) and 14-week-old BTBR$^{ob/ob}$ FMT (+) (*P*>0.99) mice. (**I**) Gene expression of podocin in the kidney showed no significant difference among 10-week-old BTBR$^{ob/ob}$ *versus* 14-week-old BTBR$^{ob/ob}$ mice FMT (-) (*P*=0.38) and 14-week-old BTBR$^{ob/ob}$ FMT (+) (*P*=0.64) mice, and between 14-week-old BTBR$^{ob/ob}$ FMT (-) and 14-week-old BTBR$^{ob/ob}$ FMT (+) (*P*>0.99) mice. (**J**) Gene expression of integrin β-1 in the kidney did not significantly change among 10-week-old BTBR$^{ob/o}$ mice *versus* 14-week-old BTBR$^{ob/ob}$ mice FMT (-) (*P*>0.99) and 14-week-old BTBR$^{ob/ob}$ FMT (+) (*P*=0.17) mice, and between 14-week-old BTBR$^{ob/ob}$ FMT(-) and 14-week-old BTBR$^{ob/ob}$ FMT (-) (*P*=0.91) mice.

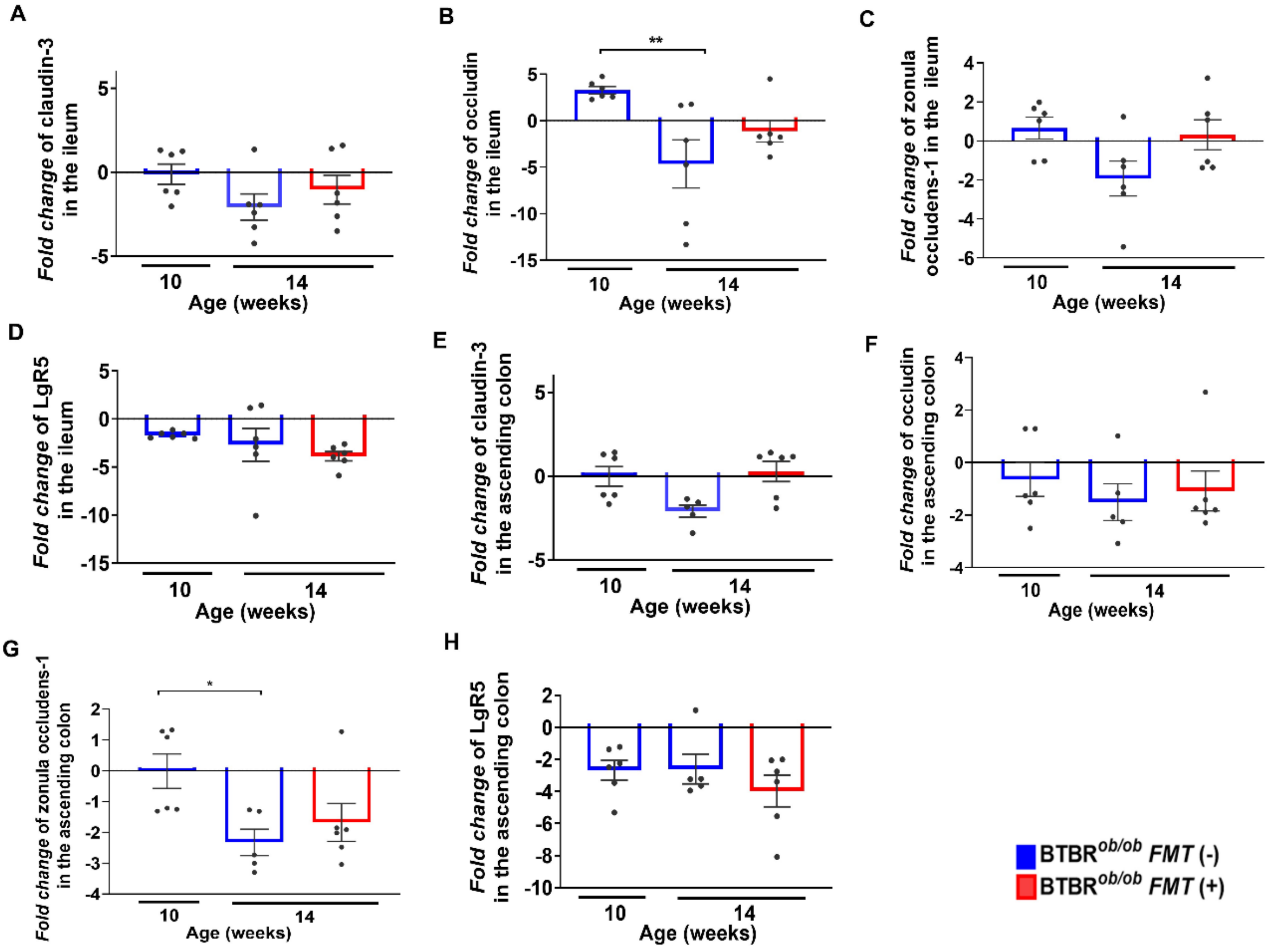

Supplementary Figure S2 . Gene expression of tight junction and stem cell marker LgR5 by qPCR in the ileum and ascending colon in BTBR$^{ob/ob}$ FMT (+) and BTBR$^{ob/ob}$ FMT (-) mice in relation to BTBR WT mice. (**A**) Gene expression of claudin-3 in the ileum of 10-week-old BTBR$^{ob/ob}$ *versus* 14-week-old BTBR$^{ob/ob}$ FMT (-) (*P*=0.19) and 14-week-old BTBR$^{ob/ob}$ FMT (+) (*P*=0.67) mice, and between 14-week-old BTBR$^{ob/ob}$ FMT (+) and 14-week-old BTBR$^{ob/ob}$ FMT (-) (*P*=0.60) mice was not different. (**B**) Gene expression of occludin in the ileum of 10-week-old BTBR$^{ob/ob}$ *versus* 14-week-old BTBR$^{ob/ob}$ FMT (-) mice was significantly different (***P*=0.009). However, occluding was not significantly different between 10-week-old BTBR$^{ob/ob}$ and 14-week-old BTBR$^{ob/ob}$ FMT (+) (*P*=0.12) mice, and between 14-week-old BTBR$^{ob/ob}$ FMT (-) and 14-week-old BTBR$^{ob/ob}$ FMT (+) (*P* >0.99) mice. (**C**) Gene expression of zonula occludens-1 in the ileum did not significantly change among 10-week-old BTBR$^{ob/o}$ *versus* 14-week-old BTBR$^{ob/ob}$ FMT (-) (*P*=0.07) and 14-week-old BTBR$^{ob/ob}$ FMT (+) (*P*=0.94) mice, and between 14-week-old BTBR$^{ob/ob}$ FMT (+) and 14-week-old BTBR$^{ob/ob}$ FMT (-) (*P*=0.12) mice. (**D**) Gene expression of LgR5 in the ileum did not significantly change among 10-week-old BTBR$^{ob/o}$ mice *versus* 14-week-old BTBR$^{ob/ob}$ FMT (-)

(P=0.76) and 14-week-old BTBR$^{ob/ob}$ FMT (+) (P=0.31) mice, and between 14-week-old BTBR$^{ob/ob}$ FMT (+) and 14-week-old BTBR$^{ob/ob}$ FMT (-) (P=0.69) mice. (**E**) Gene expression of claudin-3 in ascending colon did not significantly change among 10-week-old BTBR$^{ob/ob}$ *versus* 14-week-old BTBR$^{ob/ob}$ FMT (-) (P=0.06) and 14-week-old BTBR$^{ob/ob}$ FMT (+) (P>0.99) mice, and between 14-week-old BTBR$^{ob/ob}$ FMT (+) and 14-week-old BTBR$^{ob/ob}$ FMT (-) (P=0.06) mice. (**F**) Gene expression of occludin in ascending colon showed no significant difference among 10-week-old BTBR$^{ob/ob}$ *versus* 14-week-old BTBR$^{ob/ob}$ FMT (-) (P=0.78) and 14-week-old BTBR$^{ob/ob}$ FMT (+) (P>0.99) mice, and between 14-week-old BTBR$^{ob/ob}$ FMT (+) *versus* 14-week-old BTBR$^{ob/ob}$ FMT (-) (P>0.99) mice. (**G**) Gene expression of zonula-occludens-1 in ascending colon of 10-week-old BTBR$^{ob/ob}$ *versus* 14-week-old BTBR$^{ob/ob}$ FMT (-) mice was significantly different (*P=0.025), but was not significantly different of 14-week-old BTBR$^{ob/ob}$ mice FMT (+) (P=0.08), and between 14-week-old BTBR$^{ob/ob}$ FMT (-) and 14-week-old BTBR$^{ob/ob}$ FMT (+) (P>0.99) mice. (**H**) Gene expression of LgR5 in ascending colon of 10-week-old BTBR$^{ob/ob}$ *versus* 14-week-old BTBR$^{ob/ob}$ FMT (-) (P>0.99) and 14-week-old BTBR$^{ob/ob}$ FMT (+) (P>0.99) mice was not different, as well as between 14-week-old BTBR$^{ob/ob}$ FMT (+) and 14-week-old BTBR$^{ob/ob}$ FMT (-) (P>0.99) mice.
